# Triple Electron Attachments with a New Intermediate-Hamiltonian Fock-Space Coupled-Cluster Method

**DOI:** 10.1021/acs.jpca.4c03772

**Published:** 2024-09-13

**Authors:** Yanmei Hu, Zhifan Wang, Fan Wang, Leszek Meissner

**Affiliations:** † Institute of Atomic and Molecular Physics, Key Laboratory of High Energy Density Physics and Technology, Ministry of Education, Sichuan University, Chengdu 610065, People’s Republic of China; ‡ College of Chemistry and Life Science, Chengdu Normal University, Chengdu 611130, People’s Republic of China; § Institute of Physics, Nicholaus Copernicus University, Grudziadzka 5/7, Toruń 87-100, Poland

## Abstract

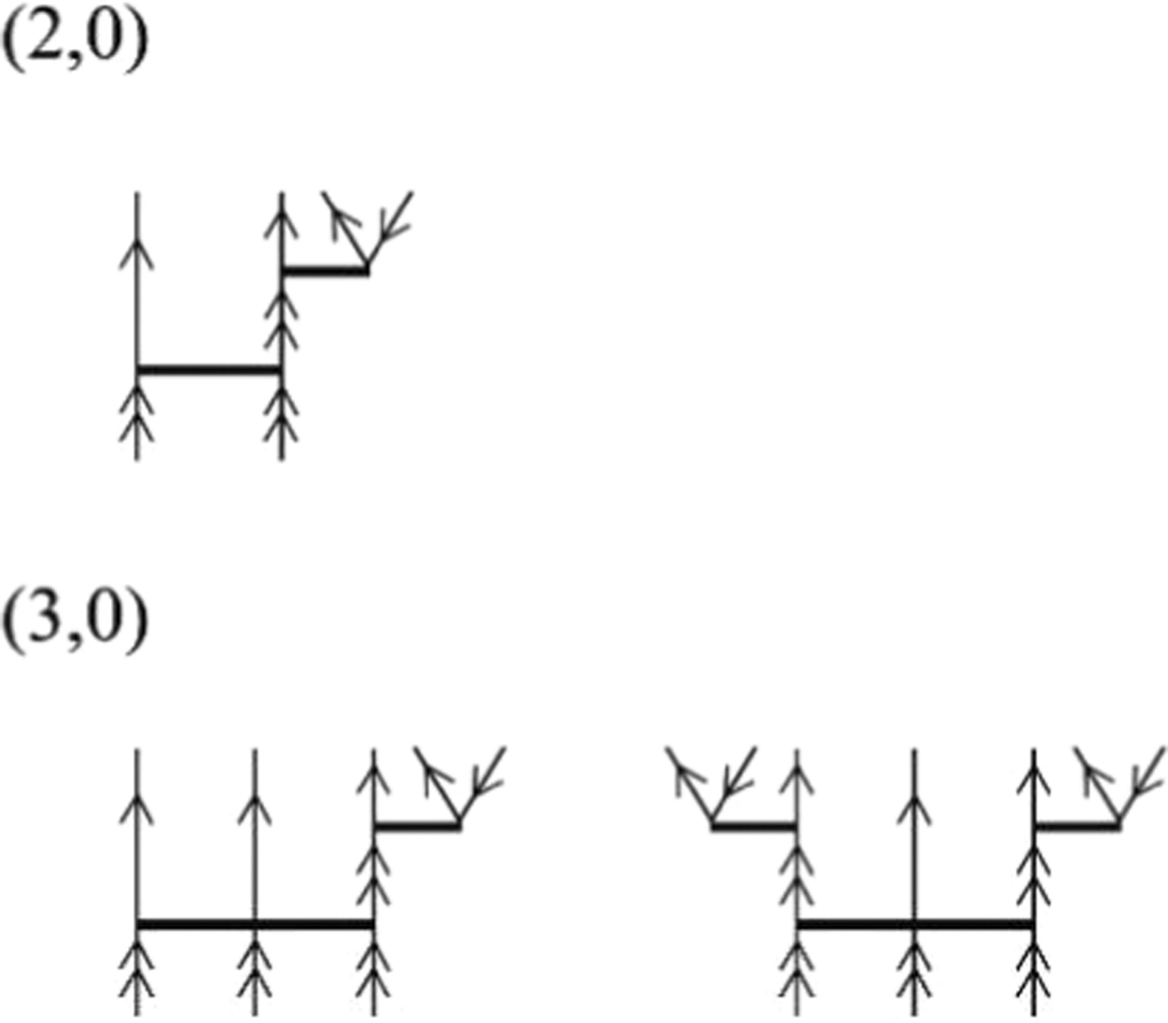

The implementation of a new intermediate-Hamiltonian Fock-space coupled-cluster (IHFSCC) scheme for the (3,0) sector of the Fock space is reported. In this IHFSCC approach, the three-body contributions in the cluster operator *S*^(3,0)^ corresponding to the (3,0) sector of the Fock space are considered, while *S*^(1,0)^ and *S*^(2,0)^ at the (1,0) and (2,0) level only include one- and two-body operators. By introducing a suitable partition of the wave operator, the intermediate Hamiltonian, which only depends on the two-body operator of S^(1,0)^, is obtained. *S*^(2,0)^ and *S*^(3,0)^ are not required within this new IHFSCC scheme, and a large reference space can be possibly employed. The performance of this (3,0) IHFSCC method in calculating triple ionization potentials and excitation energies for atoms and cations with a ground p^3^ configuration as well as adiabatic excitation energies for some molecules is investigated. The effect of the number of active virtual orbitals and three different types of orbitals, i.e., reference orbitals, restricted open-shell Hartree–Fock orbitals (ROHF) of the target systems, and canonicalized ROHF orbitals, on IHFSCC results, is also studied. Our calculations indicate that reasonable results can be achieved with this (3,0) IHFSCC method when a minimal reference space is employed. Further increasing the number of active orbitals does not necessarily improve the results. In addition, the IHFSCC results using canonicalized ROHF orbitals generally agree well with reference values, and they are not very sensitive to the number of active orbitals compared with results using the reference orbitals. The new (3,0) IHFSCC method can be applied to open-shell systems with three unpaired electrons with reasonable accuracy at a relatively low computational cost.

## I Introduction

The Fock-space coupled-cluster (FSCC)^1−6^ formalism is an important extension of the standard coupled-cluster (CC) theory developed to address problems such as excitation energies (EEs), ionization potentials (IPs), electron affinities (EAs), double ionization potentials (DIPs), and double-electron affinities (DEAs). In all of these cases, two types of electron correlation effects, i.e., dynamic and nondynamic, are present. The general idea of the FSCC approach^7−21^ is to perform a sequence of calculations starting with a closed-shell system, which can be effectively described by the single-reference CC method.^7−21^ That constitutes the calculation for the (0,0) sector of the Fock space. The Fermi vacuum used in the (0,0) sector to define cluster operators in the second-quantized form is consequently used in calculations in all other sectors of the Fock space. The presence of nondynamic correlation requires partitioning of the functional space into two subspaces: the reference space and its orthogonal complement. While the reference space is associated with the nondynamic correlation, the orthogonal space relates to the dynamic correlation effects, which are included in the calculation in an approximate manner by the so-called wave operator. The reference space functions are generated from the Fermi vacuum by a sequence of creation and annihilation operators of specially selected active spin orbitals. This selection determines reference spaces in all calculations for the sectors beyond the (0,0) one. Excitations from the reference space to its orthogonal complement are expressed as second-quantized operators defined with respect to the Fermi vacuum. In the case of the FSCC method, they are used in the exponential expansion for the wave operator. The normal-ordered form of the expansion is usually required to avoid contractions between cluster operators since they can contain particle–hole annihilation operators.

When the correlation effects for the closed-shell system are determined, the effects associated with adding or subtracting one electron can be described in the (1,0) or (0,1) sector, respectively. The calculation provides additional contributions associated with the creation or annihilation of an electron at the active orbital level. Next, the calculation for sectors with two quasi-particles can be performed, where the quasi-particle means the hole or particle created in the Fermi vacuum to obtain the reference functions. The sectors with two quasi-particles are (2,0), (0,2), and (1,1). The procedure is continued to reach the final sector describing the atomic or molecular system under consideration. Cluster amplitude equations for a given sector depend on the cluster amplitudes determined in the previous sectors, and they appear in the equations as known parameters. In this way, the knowledge about correlation effects determined in the previous sectors is transferred to the sector under consideration. In the paper, the focus is on systems that can be effectively described in the (3,0) sector; therefore, calculations for the (0,0), (1,0), and (2,0) sectors should also be considered.

There are many possible choices for the Fermi vacuum to define operators in the second-quantized form. In the case of the FSCC method, the same Fermi vacuum is used in all sectors. The choice of the Fermi vacuum depends on the investigated systems and needs to be properly made. The basic assumption is that the Fermi vacuum must correspond to a closed-shell system, which can be well described using single-reference CC methods. Besides the Fermi vacuum, the FSCC results also depend on the choice of active orbitals. IPs and EAs in the FSCC method are not affected by the active orbital selection, while EEs, DIPs, and DEAs in the FSCC method are sensitive to the choice of active orbitals.^22−26^

In the standard FSCC method, the problem of the so-called intruder states often arises, which affects the convergence of the iterative procedure used for solving the amplitude equations. Various intermediate Hamiltonian FSCC (IHFSCC) methods^27−37^ have been proposed to address this problem by introducing an intermediate space. One of the most popular IHFSCC methods is the one proposed by Meissner.^32^ The idea is to replace standard iterative procedures with diagonalization of the intermediate Hamiltonian in the space spanned by reference and intermediate space functions. That provides a powerful computational scheme for solving the equations. The results are exactly the same as those given by the standard effective Hamiltonian formulation. Diagonalization gives the energies, while cluster amplitudes can be easily obtained from the eigenvectors if they are required. A similar approach is represented by the similarity-transformed equation-of-motion coupled-cluster method (STEOM-CC).^38−45^ STEOM-CC and the IHFSCC method proposed by Meissner differ slightly in the definition of the intermediate Hamiltonian, and the results of these two methods are also rather close to each other.

The basic FSCC method includes only one- and two-particle operators in the exponent. They appear in the lowest order of perturbation expansion for the wave function since the Hamiltonian is at most a two-particle operator. If the perturbation expansion converges fast, then it provides a reliable approximation to the wave function and eigenvalues of the effective Hamiltonian. This leads to the so-called FSCC method with singles and doubles (FSCCSD).^46^ To improve the results of FSCCSD, three-particle operators can be included in the cluster operator, and this gives rise to the FSCCSDT method. Implementations of FSCC and IHFSCC at both the CCSD and the CCSDT levels have been reported.^16,47−51^ FSCCSDT or IHFSCCSDT can provide reliable results with a relatively small number of active orbitals, but the full inclusion of three-body clusters is much more expensive than FSCCSD since CCSDT equations have to be solved.^47^

Besides IPs, EAs, EEs, DIPs, and DEAs, the FSCC method can be further extended to calculate three-electron attachments (TEAs) in the (3,0) sector of the Fock space.^52^ The cluster operator in the (3,0) sector is at least a three-body operator, and such an operator is thus not included in the FSCCSD method for the (3,0) sector. The cluster operator in the (3,0) sector could possibly be important for TEAs, and one may thus include this three-body operator in the cluster operator in FSCC calculations. The IHFSCC method can be employed to eliminate this three-body operator while calculating TEAs. However, the cluster operator in the (2,0) sector of the Fock space is still required in either the FSCCSD method or the IHFSCC method. To obtain the cluster operator in the (2,0) sector of the Fock space, all double-electron-attached states corresponding to the reference space need to be obtained. This could be expensive if a relatively large reference space is employed, or the intruder-state problem may still arise even if the IHFSCC approach is adopted. A recently proposed new IHFSCC scheme for calculating TEAs addresses this issue.^53^ By redefining the wave operator and a suitable partition of the cluster operator, a new effective Hamiltonian can be introduced, which depends only on the two-electron cluster operator from the (1,0) sector of the Fock space. This new scheme provides a powerful computational scheme that can effectively deal with convergence problems in FSCC or IHFSCC, and large reference spaces can thus be employed to calculate TEAs. It should be noted that the intermediate Hamiltonian introduced in the new IHFSCC scheme is fully equivalent to that in the STEOM-CCSD method. However, the STEOM-CCSD method was not employed for TEAs.

Another popular CC approach for IPs, EAs, EEs, DIPs, and DEAs is the equation-of-motion coupled-cluster (EOM-CC)^54,55^ method. Unlike the FSCC or STEOM-CC method, the EOM-CC method is a single-reference method, and the reference space is not needed. IPs and EAs with EOM-CC are actually equivalent to those with FSCC. On the other hand, it is more expensive to calculate EEs, DIPs, and DEAs with EOM-CCSD than with FSCCSD or IHFSCCSD. The EOM-CCSD results for TEAs have also been reported; however, they are calculated only within the 3p space. The error of such a method could be rather pronounced.^56^ The computational scaling will be O(N^7^) if the 4p1h determinants are to be considered, and that would be too expensive. On the other hand, the computational cost of the IHFSCC method for TEAs is much more modest, and the method can be easily applied to larger systems or systems with a large basis set.

In this work, we report the implementation of the new IHFSCC method^53^ for TEAs. Triple ionization potentials and excitation energies for atoms or ions with a ground p^3^ configuration as well as those for molecules with a σ^1^π^2^ configuration are presented. In the calculations, three types of orbitals are employed to construct the reference determinant: the Hartree–Fock (HF) orbitals of the closed-shell reference state with three electrons less, the restricted open-shell HF (ROHF) orbitals of the target state, and the canonical orbitals from ROHF orbitals. The effect of different sizes of the reference space on TEAs using these three types of orbitals is also investigated. This paper is organized in the following manner: basic theory and implementation details are presented in Section II; results of the new IHFSCC method using different orbitals and different reference spaces are given and compared with the available experimental data and other theoretical results in Section III. Conclusions are drawn in Section IV.

## II Theory and Implementation Details

The wave operator in the (3,0) sector of the FSCC calculations takes the following form: Ω = *e*^*T*^{*e*^*S̃*(3,0)^}*P*, where *T* is the cluster operator in the single-reference CC method used in the (0,0) sector of the Fock space, *S̃*^(3,0)^ is the cluster operator in the (3,0) sector of the Fock space, *P* is the projector on the (3,0) reference space, and {} stands for the normal-order form. *S̃*^(3,0)^ can be partitioned according to the number of annihilation operators of active particles: *S̃*^(3,0)^ = *S*^(1,0)^ + *S*^(2,0)^ + *S*^(3,0)^, where *S*^(*k*,0)^ stands for cluster operators containing *k* annihilation operators of active particles and takes the following form
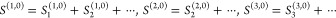
1
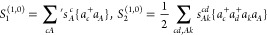
2
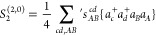
3
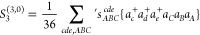
4In the above equations, the subscript in *S*_*n*_^(*k*,0)^ denotes the particle rank of the corresponding cluster operator, *A* and *B···* are indices for active virtual orbitals, *k* and *l*··· are indices of occupied orbitals, and *c* and *d*,··· are those of unoccupied ones. The prime in the summations indicates that excitations within the reference space are excluded. The amplitudes in *S*^(3,0)^ operators can be determined from the following Bloch equation:
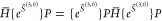
5where *H̅* stands for similarity-transformed Hamiltonian *e*^–*T*^*He*^*T*^ – *E*_CC_, from which the (0,0) energy *E*_CC_ is subtracted. It should be noted that *S*^(*k*,0)^ for *k* < 3 is determined using the Bloch equation in the (*k*,0) sectors of the Fock space. When *S̃*^(3,0)^ is determined, energies of triple-electron-attached states are given by the eigenvalues of the effective Hamiltonian *PH̅*{*e*^*S̃*^(3,0)^^}*P*.

At the CCSD level, where only one- and two-particle operators are considered in *S*^(*k*,0)^, *S*^(3,0)^ is not included, since it is at least a three-particle operator. It might be noted that *S*^(1,0)^ and *S*^(2,0)^ are calculated by solving the Bloch equation in the (1,0) and (2,0) sectors of the Fock space, respectively. TEAs can readily be obtained when *S*^(1,0)^ and *S*^(2,0)^ are available. This method has been reported previously.^52^ Contribution of *S*^(3,0)^ could possibly be important for states in the (3,0) sector of Fock space. One can include *S*_3_^(3,0)^ in *S̃*^(3,0)^ and calculate *S*_3_^(3,0)^ by solving eq 5.

Solving the Bloch equation often suffers from the intruder-state problem. In the IHFSCC method for the (*k*,0) sector of the Fock space proposed by Meissner,^32^ the intermediate space is defined by the action of the *S*^(*k*,0)^ on the reference space. The {*e*^*S̃*^(*k*,0)^^}*P* operator is written as {*e*^*S̃*^(*k*,0)^^}*P* = (1 + *Y*^(*k*,0)^ + *Z*^(*k*,0)^)*P*, where *Z*^(*k*,0)^ contains operators producing determinants in the intermediate space and *Y*^(*k*,0)^ contains operators generating determinants outside the intermediate space while acting on the corresponding reference space. The intermediate Hamiltonian *P*_1_*H̅*(1 + *Y*^(*k*,0)^)*P*_1_ for the (*k*,0) sector of the Fock space, which does not depend on *S*^(*k*,0)^, is introduced, where *P*_1_ is the projector on the union of the model space and the intermediate space. The intermediate Hamiltonian is shown to have the same eigenvalues as the effective Hamiltonian in the standard FSCC method for eigenvectors located mainly in the model space.^32^ When *S*_3_^(3,0)^ is introduced in the cluster operator, then the *Y*^(3,0)^ operator takes the following form:
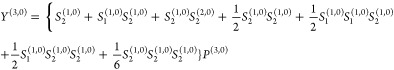
6Energies of triple electron attachment states can be obtained from the eigenvalues of *P*_1_*H̅*(1 + *Y*^(3,0)^)*P*_1_, where *S*_3_^(3,0)^ does not need to be calculated. No matter whether *S*_3_^(3,0)^ is included in *S̃*^(3,0)^, *S*^(1,0)^ and *S*^(2,0)^ are always required to construct the effective Hamiltonian. *S*^(1,0)^ can be obtained from the eigenvectors R in the EOM-EA-CCSD calculations for electron-attached states corresponding to active virtual orbitals with the following equation:
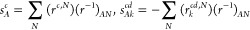
7where *r* are the *R*-vector coefficients and *N* in the above equations is the index for electron-attached states. On the other hand, all double-electron-attached states in the active space have to be calculated to obtain *S*^(2,0)^. This could be expensive when a large number of active virtual orbitals are chosen. In addition, some double-electron-attached states may not have a dominant contribution from the reference space, which is closely related to the intruder-state problem.

To avoid calculating *S*^(2,0)^ in the (3,0) sector FSCC calculations, the wave operator can be slightly modified
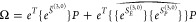
8
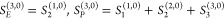
9Here, the standard normal-ordered exponential expansion for the wave operator is replaced by a product of two normal-ordered exponential expansions. The first one involves *S_E_*^(3,0)^, while the second one has *S_P_*^(3,0)^ as an exponent. As a consequence, contractions between *S_E_*^(3,0)^ and *S_P_*^(3,0)^ are possible, and the additional term appears in the wave operator, which contains at least one contraction between *S_E_*^(3,0)^ and *S_P_*^(3,0)^. Now, a new *Y*^(3,0)^ operator, which acts on the space spanned by all determinants with three electrons on unoccupied orbital levels, can be introduced: *Y*^(3,0)^ = {*e*^*S_E_*^(3,0)^^ – 1}*P*_I_. This new approach allows us to omit calculations at the (2,0) level in the valence-universal strategy since energies for states in the (3,0) sector can be obtained from the eigenvalues of the intermediate Hamiltonian *P*_1_*H̅*{*e*^*S*_2_^(1,0)^^}*P*_1_, which does not depend on *S*_2_^(2,0)^. This new IHFSCC scheme can also be applied to other sectors of the Fock space. The detailed derivation for the new IHFSCC scheme can be found in ref (53). It should be noted that this new intermediate Hamiltonian becomes exactly the same as that in STEOM-CCSD. Compared with STEOM-CCSD, both the operator used in the similarity transformation of the Hamiltonian and its inverse in IHFSCC are well defined. Moreover, the negligence of some categories of clusters can be theoretically justified by introducing a sequence of similarity transformations, which introduces contractions between two types of cluster operators.

This new (3,0) sector IHFSCC approach is implemented using our previous implementation of the (2,0) sector IHFSCC method^57^ through an interface to the CFOUR^58^ and PySCF program packages,^59^ where orbital energies and two-electron integrals in molecular orbital basis are obtained. The following steps are performed to obtain TEAs with the new (3,0) sector IHFSCC method: (1) the CCSD calculation to obtain the T operator, (2) calculating one- and two-electron parts of the transformed Hamiltonian *e*^*–T*^*He*^*T*^ – *E*_CC_, (3) choosing active virtual orbitals and performing EOM-EA-CCSD calculations for electron-attached states of these active orbitals, (4) calculating S_2_^(1,0)^ form R-vector of EOM-EA-CCSD with eq 7 and constructing the one- and two-electron parts of the intermediate Hamiltonian, and (5) calculating eigenvectors and eigenvalues of the intermediate Hamiltonian in 3p space. All of these five steps are performed with programs developed in our group. Davidson’s algorithm with a slight modification for non-Hermitian matrix^60,61^ is employed in step (5) to obtain the lowest eigenvalues and the corresponding eigenvectors of the intermediate Hamiltonian. Detailed algebraic expressions for the one- and two-electron parts of the intermediate Hamiltonian and the multiplication between the intermediate Hamiltonian and a trial vector are given in the Appendix. In Davidson’s algorithm, diagonal elements of the intermediate Hamiltonian are required to calculate the error vector to be supplemented to the existing subspace.^60,61^ In our previous implementations in EOM-CCSD or IHFSCC calculations,^57,62−66^ we always use orbital energy differences to approximate the diagonal elements and do not have a problem with this approximation. However, such an approximation would lead to a convergence problem in calculating TEAs. This may be because orbital energy differences differ significantly from the diagonal elements for TEAs. In this work, the contribution of the <vv||vv> integrals to the diagonal elements is also considered, and converged results can be achieved.

In our calculations, the reference state is always a closed-shell state, and the final three-electron-attached states are either doublet states or quadruple states. In our implementation, we always calculate states with *M*_s_ = 1/2, i.e., eigenvectors with the spin case of *R*^*abc̅*^, where indices with a bar are for α spin orbitals. Spatial symmetry of Abelian subgroup is exploited, and the direct-product decomposition (DPD) method^67^ is employed to deal with spatial symmetry. In (3,0) IHFSCC calculations, the reference states have three electrons less than the three-electron-attached states. Orbital relaxation effects could be significant when orbitals of the reference state are employed. Three types of orbitals are adopted in the present work in calculating TEAs: the reference orbitals, the restricted open-shell Hartree–Fock (ROHF) orbitals for the ground three-electron-attached state, and canonicalized ROHF orbitals. In obtaining canonicalized ROHF orbitals, the occupied–occupied and virtual–virtual parts of the Fock matrix for the reference state using ROHF orbitals are diagonalized. Reference orbitals and corresponding two-electron integrals are calculated with CFOUR, while ROHF orbitals and canonicalized ROHF orbitals as well as two-electron integrals in these orbitals are obtained from PySCF. It should be noted that the Fock matrix employed in CCSD, EOM-EA-CCSD, and IHFSCC calculations is no longer diagonal with ROHF or canonicalized ROHF orbitals. CCSD energies and EAs with EOM-EA-CCSD using ROHF orbitals are the same as those with canonicalized ROHF orbitals, since they are invariant with respect to unitary transformation within occupied or virtual orbital space. However, this is not the case for IHFSCC results since the active space with ROHF orbitals is not the same as that with canonicalized ROHF orbitals.

## III Computational Details and Results

The new (3,0) sector IHFSCCSD method is applied to triple ionization energies (TIPs) and EEs of atoms or cations with a ground p^3^ configuration of the first and second row elements, i.e., N, O^+^, F^2+^, Ne^3+^, P, S^+^, Cl^2+^, and Ar^3+^. EEs for excited states originating from the np^3^ and np^2^(*n* + 1)s^1^ configurations of these atoms and cations are calculated. In these calculations, various p and s orbitals are chosen as the active orbitals. We note that triple ionization potentials and excitation energies for states from the np^3^ configuration using only np orbitals as active resemble closely those using np and (*n* + 1)s orbitals as active space. We are interested in both np^3^ and np^2^(*n* + 1)s^1^ configurations, and the results using np orbitals as active orbitals are thus not reported. Besides these atoms or cations, equilibrium geometries, harmonic frequencies, and adiabatic excitation energies of X ^4^∑^–^ and ^2^Π states for LiC and NaC and adiabatic excitation energies of CH and NH^+^ are also calculated with the new IHFSCC method. The ground state with the σ^1^π^2^ configuration and the excited state from the σ^2^π^1^ configuration are calculated for LiC and NaC. As for CH and NH^+^, the ground-state configuration of these molecules is σ^2^π^1^, and EEs of the states from the σ^1^π^2^ configuration are calculated. Equilibrium geometries and harmonic frequencies of LiC and NaC are obtained by fitting the total energies of the seven points around equilibrium bond lengths with a space of 0.05 Å using a polynomial up to fifth order. On the other hand, experimental bond lengths^68^ are employed for different states in CH and NH^+^.

In all of these calculations, the closed-shell state with fewer than three electrons is chosen as the reference. The aug-cc-pVQZ basis set^69^ is employed except for N and P, and all electrons are correlated in our calculations for atoms. The d-aug-cc-pVQZ basis set^70^ is adopted for N and P since additional diffuse basis functions are important for their np^2^(*n* + 1)s^1^ configuration. We note that the additional diffuse basis function has negligible effects on the np^3^ configuration of these two atoms and on the other ions. In addition, EOM-TEA-CCSD calculations are also performed, where TEAs are obtained from eigenvalues of the effective Hamiltonian *e*^–*T*^*H*e^*T*^ in 3p space. Experimental data,^71^ available CCSD(T), and previous FSCCSD(2)^52^ results are also given for comparison. FSCCSD(2) is an approximate FSCCSD method proposed in ref (52) where a three-particle component of the (3,0) effective Hamiltonian is reduced to second-order contributions. In CCSD(T) calculations for open-shell states, unrestricted HF orbitals are employed. On the other hand, the ^2^D states of the p^3^ configuration are also calculated with a CCSD(T) program developed in our group with complex orbitals using determinant |p_1_αp_1_βp_0_α| as the reference. To facilitate comparison with previous FSCC results,^52^ the aug-cc-pVTZ basis set^69^ is used for molecules. All calculations in this work were performed in a Hefei advanced computing center.

### III.I Triple Ionization Potentials for Atoms or Cations

TIPs for the atoms or cations with the new IHFSCC method using different orbitals and selecting different active orbitals as well as those obtained with EOM-CCSD and CCSD(T) are listed in Table 1, together with the available experimental data.^71^ Mean error (ME), mean absolute error (MAE), maximum error (MAX), minimum error (MIN) and standard deviation (SD) of IHFSCC and TEA-EOM-CCSD results compared with experimental data or CCSD(T) results are also given in the table. In the tables, the following notation is used: orbit1 denotes reference orbitals, orbit2 represents canonicalized ROHF orbitals, and orbit3 denotes ROHF orbitals. One can see from the table that TIPs with CCSD(T) are about 0.2–0.3 eV smaller than experimental data except for Cl^2+^ and Ar^3+^. Considering the fact that TIPs for these atoms or cations amount to 60–300 eV, CCSD(T) can achieve reliable TIPs for these atoms and cations. As for Cl^2+^ and Ar^3+^, CCSD(T) underestimate their TIPs by about 0.6 and 1.1 eV, respectively. Our test calculations indicate that relativistic effects^72,73^ increase TIPs for these two cations by about 0.1 eV, and the use of the aug-cc-pV5Z^69^ basis set can further increase their TIPs by about 0.15 eV. On the other hand, the uncertainty of experimental TIPs for these two cations is about 0.3–0.4 eV.^71^ We note that the use of a larger basis set has almost no effect on EEs for the investigated systems.

**Table 1:** Triple Ionization Potentials (TIP) in eV for Atoms or Cations with a p^3^ Configuration Using CCSD(T), TEA-EOM-CCSD, and IHFSCC with Difference Orbitals and Active Space^a^

						number of active orbitals:4			number of active orbitals:8			number of active orbitals:11		
atom	state		expt^b^	CCSD(T)	TEA-EOM-CCSD	orbit1	orbit2	orbit3	orbit1	orbit2	orbit3	orbit1	orbit2	orbit3
N	2s^2^2p^3^	^4^S°	91.58	91.45	85.46	91.24	91.58	91.75	90.49	91.27	91.22	90.92	91.38	91.39
O^+^	2s^2^2p^3^	^4^S°	167.47	167.27	160.44	167.03	167.34	167.45	166.27	167.06	167.05	166.41	167.08	167.12
F^2+^	2s^2^2p^3^	^4^S°	264.13	263.86	256.51	263.62	263.89	263.96	262.84	263.64	263.75	263.39	263.76	
NE^3+^	2s^2^2p^3^	^4^S°	381.37	381.06	373.36	380.82	381.07	381.12	380.02	380.83	380.91	380.03	380.87	380.9
P	3s^2^3p^3^	^4^S°	60.46	60.25	56.63	59.88	60.31	60.41	59.89	60.16	60.53	59.71	60.10	60.12
S^+^	3s^2^3p^3^	^4^S°	105.42	105.23	101.14	105.09	105.25	105.31	104.85	105.10	105.29	105.02	105.12	105.13
CL^2+^	3s^2^3p^3^	^4^S°	160.72	160.11	155.68	159.97	160.11	160.16	159.7	159.93	159.99	159.79	159.93	159.95
AR^3+^	3s23p3	4S°	225.71	224.62	219.92	224.48	224.6	224.64	224.43	224.56	224.58	224.17	224.58	224.58
Expt		ME			–5.97	–0.59	–0.34	–0.26	–1.05	–0.54	–0.44	–0.93	–0.50	–0.51
		MAE			5.97	0.59	0.34	0.30	1.05	0.54	0.46	0.93	0.50	0.51
		MIN			–8.01	–1.23	–1.11	–1.07	–1.35	–1.15	–1.13	–1.54	–1.13	–1.13
		MAX			–3.83	–0.33	0.00	0.17	–0.57	–0.30	0.07	–0.40	–0.20	–0.19
		SD			1.43	0.27	0.34	0.36	0.29	0.28	0.34	0.35	0.29	0.29
CCSD(T)		ME			–5.59	–0.22	0.04	0.12	–0.67	–0.16	–0.07	–0.55	–0.13	–0.11
		MAE			5.59	0.22	0.04	0.12	0.67	0.16	0.15	0.55	0.13	0.11
		MIN			–7.70	–0.37	–0.02	0.02	–1.04	–0.23	–0.23	–1.03	–0.19	–0.16
		MAX			–3.62	–0.14	0.13	0.30	–0.19	–0.06	0.28	–0.21	–0.04	–0.04
		SD			1.48	0.07	0.04	0.09	0.34	0.06	0.16	0.25	0.05	0.04

a Mean error (ME), mean absolute error (MAE), maximum error (MAX), minimum error (MIN), and standard deviation (SD) compared with experimental data and CCSD(T) results are also given. (basis set: aug-cc-pVQZ).

b Reference (71)

According to Table 1, TEA-EOM-CCSD underestimated TIPs of these systems by about 4–8 eV since contributions from 4p1h determinants are not considered, while TIPs with the new IHFSCC method are improved pronouncedly. One can see from the table that TIPs with canonicalized ROHF orbitals agree well with those using ROHF orbitals except for N with 4 active orbitals and P with 8 active orbitals, while TIPs using reference orbitals are somewhat smaller than those using ROHF or canonicalized ROHF orbitals. The large difference between results using canonicalized ROHF orbitals and ROHF orbitals for N and P stems from the fact that more than one orbitals contribute significantly to some electron-attached states with EOM-EA-CCSD using ROHF orbitals. This makes choosing a proper active space nonstraightforward with ROHF orbitals. On the other hand, this is not the case with canonicalized ROHF orbitals or reference orbitals. We can also see that TIPs are generally reduced when the number of active orbitals increases from 4 to 8. On the other hand, TIPs with 11 active orbitals are usually between those with 8 active orbitals and those with 4 active orbitals. The number of active orbitals has a significant effect on TIPs using the reference orbitals, while their effects are more modest with ROHF or canonicalized ROHF orbitals. According to Table 1, TIPs with canonical ROHF orbitals and 4 active orbitals agree the best with the experimental data or CCSD(T) results. This is probably due to a more balanced error cancellation with this orbital and active orbital selection.

### III.II Excitation Energies of Atoms or Cations

EEs for states from the np^3^ configuration of these atoms and cations are listed in Table 2, and those for states from the np^2^(*n* + 1)s^1^ configuration are given in Table 3. The performance of the new IHFSCC method using various orbitals and active space is different for these two configurations, and their EEs are thus listed separately. CCSD(T) results for the ^2^D state from np^3^ configuration and the ^4^P state from np^2^(*n* + 1)s^1^ configuration, TEA-EOM-CCSD results, experimental data,^71^ and as well as previous FSCCSD(2)^52^ results using 4 active orbitals are also listed for comparison. ME, MAE, MAX, MIN, and SD of EEs with these methods compared with experimental data are presented at the bottom of these two tables. It can be seen from Table 2 that EEs of states from p^3^ configuration with CCSD(T) are only about 0.03–0.05 eV larger than experimental data. TEA-EOM-CCSD can provide reasonable EEs for the ^2^D states, but it underestimates the EEs of the ^2^P state pronouncedly. On the other hand, reliable EEs of p^3^ configuration are obtained with the new IHFSCC method. Results of the new IHFSCC method for these EEs are not sensitive to the employed orbitals and active space, and their MAEs are 0.11–0.15 eV. Considering the fact that the computational cost of IHFSCC depends on the size of the active space, using 4 active orbitals can already provide reliable EEs for p^3^ configuration with the IHFSCC method. On the other hand, EEs of FSCCSD(2)^52^ are generally larger than results of the new IHFSCC method using 4 active orbitals and reference orbitals, but their MAEs are quite close to each other. It is unclear whether this difference is due to the inclusion of S(3,0) in the new IHFSCC method or to approximations introduced in FSCCSD(2).

**Table 2:** Excitation Energies (EEs) in eV for States in np^3^ Configuration with TEA-EOM-CCSD and IHFSCC Using Different Orbitals and Active Space^a^

					number of active orbitals:4			number of active orbitals:8			number of active orbitals:11			FSCCSD(2)^c^
atom	state		reference^b^	TEA-EOM-CCSD	orbit1	orbit2	orbit3	orbit1	orbit2	orbit3	orbit1	orbit2	orbit3	orbit1
N	2s^2^2p^3^	^2^D°	2.38(2.42)	2.23	2.27	2.34	2.32	2.3	2.22	2.2	2.32	2.24	2.25	2.34
		^2^P°	3.58	2.73	3.46	3.47	3.50	3.52	3.37	3.35	3.58	3.40	3.41	3.58
O^+^	2s^2^2p^3^	^2^D°	3.33(3.38)	3.28	3.23	3.30	3.29	3.24	3.17	3.16	3.22	3.15	3.17	3.47
		^2^P°	5.02	4.04	4.79	4.83	4.86	4.82	4.72	4.72	4.82	4.72	4.74	4.95
F^2+^	2s^2^2p^3^	^2^D°	4.23(4.28)	4.21	4.16	4.21	4.21	4.14	4.22	4.12	4.02	4.19	4.09	4.36
		^2^P°	6.39	5.26	6.07	6.16	6.16	6.10	6.16	6.07	5.96	6.19	6.11	6.18
NE^3+^	2s^2^2p^3^	^2^D°	5.12(5.17)	5.12	5.07	5.12	5.12	5.04	4.97	5.01	5.03	4.97	4.99	5.11
		^2^P°	7.74	6.49	7.33	7.42	7.44	7.36	7.30	7.34	7.38	7.33	7.34	7.36
P	3s^2^3p^3^	^2^D°	1.41(1.43)	1.49	1.34	1.35	1.33	1.39	1.35	1.32	1.39	1.34	1.35	1.38
		^2^P°	2.32	1.93	2.45	2.42	2.44	2.46	2.39	2.44	2.42	2.36	2.37	2.57
S^+^	3s^2^3p^3^	^2^D°	1.84(1.88)	2.01	1.76	1.77	1.76	1.81	1.77	1.75	1.81	1.77	1.78	
		^2^P°	3.04	2.59	3.16	3.15	3.17	3.15	3.10	3.16	3.15	3.11	3.11	
CL^2+^	3s^2^3p^3^	^2^D°	2.24(2.29)	2.46	2.16	2.17	2.16	2.20	2.17	2.17	2.20	2.17	2.17	
		^2^P°	3.70	3.17	3.82	3.82	3.83	3.78	3.74	3.77	3.77	3.74	3.75	
AR^3+^	3s^2^3p^3^	^2^D°	2.62(2.65)	2.88	2.54	2.55	2.54	2.52	2.51	2.52	2.54	2.56	2.54	
		^2^P°	4.33	3.73	4.45	4.44	4.45	4.54	4.44	4.45	4.56	4.50	4.48	
ME				–0.35	–0.08	–0.05	–0.04	–0.06	–0.11	–0.09	–0.07	–0.10	–0.10	–0.02
MAE				0.45	0.14	0.11	0.11	0.12	0.14	0.15	0.13	0.14	0.14	0.13
MIN				–1.25	–0.41	–0.32	–0.30	–0.38	–0.44	–0.40	–0.43	–0.41	–0.40	–0.38
MAX				0.26	0.13	0.12	0.13	0.21	0.11	0.12.	0.23.	0.17	0.15	0.13
SD				0.48	0.15	0.12	0.12	0.15	0.14	015	0.16	0.14	0.14	0.14

a The reference values are experimental data, and CCSD(T) results are given in parentheses. Mean error (ME), mean absolute error (MAE), maximum error (MAX), minimum error (MIN), standard deviation (SD) compared with experimental data are also given (basis set: aug-cc-pVQZ).

b Reference^71^.

c Reference^52^

**Table 3:** Excitation Energies (EEs) in eV for States in np^2^(*n* + 1)s^1^ Configuration with TEA-EOM-CCSD and IHFSCC Using Different Orbitals and Active Space^a^

					number of active orbitals:4			number of active orbitals:8			number of active orbitals:11			FSCCSD(2)^c^
atom	state		reference^b^	TEA-EOM-CCSD	orbit1	orbit2	orbit3	orbit1	orbit2	orbit3	orbit1	orbit2	orbit3	orbit1
N	2s^2^2p^2^(^3^P)3s	^4^P	10.33(10.32)	8.48	10.15	10.35	10.48	10.09	10.52	10.47	10.42	10.65	10.67	10.67
		^2^P	10.68	8.77	10.44	10.64	10.78	10.31	10.85	10.81	10.71	11.00	10.99	
	2s^2^2p^2^(^1^D)3s	^2^D	12.36	10.39	12.04	12.29	12.42	11.95	12.41	12.36	12.30	12.56	12.56	
O^+^	2*s*^2^2*p*^2^(^3^P)3s	^4^P	22.98(22.99)	20.21	22.64	22.84	22.8	22.23	22.87	22.85	22.23	22.90	22.92	
		^2^P	23.43	20.60	23.24	23.48	23.48	22.72	23.12	23.11	22.72	23.16	23.18	
	2s^2^2p^2^(^1^D)3s	^2^D	25.66	22.79	25.24	25.50	25.46	24.78	25.35	25.34	24.78	25.38	25.41	
	2s^2^2p^2^(^1^S)3s	^2^S	28.59	24.06	27.84	28.10	28.11	27.39	27.88	27.87	27.39	27.91	27.96	
F^2+^	2s^2^2p^2^(^3^P)3s	^4^P	39.30(39.34)	36.27	38.96	39.09	39.09	38.57	39.35	39.16	39.11	39.16	39.4	39.20
		^2^P	40.26	36.83	39.84	40.05	40.05	39.32	40.30	40.10	39.72	39.98	40.12	40.30
	2s^2^2p^2^(^1^D)3s	^2^D	42.65	39.51	42.25	42.44	42.44	41.81	42.70	42.48	42.25	42.45	42.58	42.69
	2s^2^2p^2^(^1^S)3s	^2^S	46.21	41.24	45.39	45.64	45.64	44.96	45.86	45.64	45.35	45.64	46.64	45.73
NE^3+^	2s^2^2p^2^(^3^P)3s	^4^P	59.41(59.52)	56.27	59.11	59.29	59.22	58.85	59.38	59.45	58.85	59.41	59.43	
		^2^P	60.61	57.03	60.29	60.52	60.49	59.81	60.58	60.64	59.83	60.59	60.61	
	2s^2^2p^2^(^1^D)3s	^2^D	63.45	60.17	63.09	63.32	63.26	62.76	63.36	63.44	62.76	63.38	63.41	
	2s^2^2p^2^(^1^S)3s	^2^S	67.66	62.39	66.78	67.05	67.02	66.44	67.07	67.15	66.46	67.11	67.14	
P	3s^2^3p^2^(^3^P)4s	^4^P	6.96(6.95)	6.00	6.86	6.96	7.02	6.94	6.94	6.91	6.82	6.90	7.02	
		^2^P	7.19	6.26	7.09	7.20	7.27	7.16	7.15	7.12	7.01	7.11	7.29	
	3s^2^3p^2^(^1^D)4s	^2^D	8.08	7.17	7.89	8.00	8.06	7.99	7.97	7.94	7.86	7.93	8.07	
	3s^2^3p^2^(^1^S)4s	^2^S	9.54	7.86	9.57	9.64	9.72	9.63	9.55	9.51	9.47	9.50	9.57	
S^+^	3s^2^3p^2^(^3^P)4s	^4^P	13.62(13.62)	12.40	13.48	13.59	13.53	13.47	13.59	13.66	13.61	13.62	13.62	
		^2^P	14.04	12.69	13.88	13.98	13.94	13.78	13.96	13.95	13.93	14.00	13.99	
	3s^2^3p^2^(^1^D)4s	^2^D	15.07	13.88	14.83	14.94	14.87	14.81	14.94	14.97	14.94	14.97	14.97	
	3s^2^3p^2^(^1^S)4s	^2^S	16.90	14.75	16.86	16.92	16.88	16.74	16.84	16.95	16.87	16.88	16.88	
CL^2+^	3s^2^3p^2^(^3^P)4s	^4^P	21.59(21.68)	20.42	21.50	21.56	21.51	21.45	21.46	21.54	21.52	21.50	21.52	
		^2^P	22.16	20.84	22.15	22.26	22.21	22.03	22.19	22.25	22.12	22.22	22.23	
	3s^2^3p^2^(^1^D)4s	^2^D	23.36	22.17	23.21	23.24	23.19	23.19	23.26	23.28	23.28	23.27	23.28	
Ar^3+^	3s^2^3p^2^(^3^P)4s	^4^P	31.12	29.58	31.01	31.09	31.04	31.13	31.19	31.20	30.85	31.18	31.19	
		^2^P	31.83	29.83	31.68	31.79	31.75	31.71	31.8	31.81	31.57	31.56	31.69	
	3s^2^3p^2^(^1^D)4s	^2^D	33.20	30.74	32.94	33.02	32.97	32.98	33.03	33.05	32.75	33.14	33.08	
ME				–2.36	–0.28	–0.12	–0.12	–0.46	–0.10	–0.06	–0.37	–0.11	–0.03	–0.04
MAE				2.36	0.28	0.14	0.17	0.46	0.14	0.12	0.38	0.18	0.15	0.20
MIN				–5.27	–0.88	–0.61	–0.64	–1.25	–0.71	–0.57	–1.2	–0.68	–0.63	–0.48
MAX				–0.91	0.03	0.10	0.18	0.09	0.19	0.19	0.09	0.32	0.43	0.03
SD				1.19	0.22	0.17	0.19	0.40	0.20	0.17	0.36	0.22	0.21	0.15

a The reference values are experimental data, and CCSD(T) results are given in parentheses. Mean error (ME), mean absolute error (MAE), maximum error (MAX), minimum error (MIN), and standard deviation (SD) compared with experimental data are also given (basis set: aug-cc-pVQZ).

b Reference (71).

c Reference (52)

As for EEs of states from the np^2^(*n* + 1)s^1^ configuration, we note that we are not able to obtain the ^2^S state for N since many lower states exist with the d-aug-cc-pVQZ basis set. In addition, there are no available experimental data for ^2^S states of Cl^2+^ and Ar^3+^, and these states are thus not reported in Table 3. In addition, the ^4^P state of Ar^3+^ cannot be obtained with CCSD(T) due to a strong mixing between the 3d and 4s orbitals. One can see from Table 3 that CCSD(T) can provide reliable EEs for the ^4^P state with an error of less than 0.1 eV, while TEA-EOM-CCSD underestimates them significantly. The new IHFSCC method can provide reasonable EEs for states from np^2^(*n* + 1)s^1^ configuration, and their error is somewhat larger than those for np^3^ states, especially when the reference orbitals are adopted. Similar to the case for the np^3^ configuration, the results using ROHF orbitals are rather close to those using canonicalized ROHF orbitals in most cases. EEs with reference orbitals for these states are generally smaller than those using ROHF or canonicalized ROHF orbitals. On the other hand, the MAE of these EEs with IHFSCC is about 0.28 eV with the reference orbitals using 4 active orbitals, and it is even larger when a larger active space is adopted. A similar situation also occurs for the maximum absolute errors with the reference orbitals. According to EEs in these two tables, one can see that reliable results can be obtained with the new IHFSCC method using canonicalized ROHF orbitals with 4 active orbitals.

IHFSCC or STEOM-CC results are closely related to the percentage of the final eigenstate in the active space (active%) as well as to the percentage of single excitations (*R*_1_%) of EOM-EA-CCSD wave function for the electron-attached state. Our previous IHFSCCSD results for the (1,1), (2,0), and (0,2) sectors^52^ show that active% increases and IHFSCC results will be improved with a larger active space when *R*_1_% for the active orbitals are large. However, this is not the case for TEAs with the new (3,0) sector IHFSCC method. According to our results, *R*_1_% for the involved electron-attached states is always larger than 95% with 4 active orbitals. Active% for states from the np^3^ configuration range from 70 to 95% with reference orbitals or canonical ROHF orbitals for states from the np^3^ configuration. On the other hand, they are quite close to 100% with ROHF orbitals. However, the results of the new IHFSCC method with canonical ROHF orbitals for the np^3^ configuration are quite close to those with ROHF orbitals in most cases. As for states from the np^2^(*n* + 1)s^1^ configuration, active% with the reference orbitals or the canonical ROHF orbitals are even smaller; reasonable results with the new IHFSCC method can still be achieved, especially with the canonical ROHF orbitals. When more virtual orbitals are included as active orbitals, *R*_1_% for some electron-attached states is indeed not very large. On the other hand, active% is around 97–98% for most of the states with all of these orbitals. However, results of the new IHFSCC method are not improved with a larger active space even for systems with *R*_1_% always larger than 95%. According to our results, using a minimum active space with the canonical ROHF orbitals can already provide reasonable results with the new (3,0) sector IHFSCC method.

As for he effects of orbitals on the results of the new (3,0) sector IHFSCC method, orbital relaxation is expected to be pronounced with the reference orbitals, while such effects could be reduced with ROHF or canonical ROHF orbitals of the target ground state. However, the error of the CCSD method on the reference with ROHF or canonical ROHF orbitals will be larger. Fortunately, orbital relaxation effects can be reliably described with CCSD through a single excitation operator in the exponential. This could possibly explain why the results of the new (3,0) sector IHFSCC method with ROHF or canonical ROHF orbitals are generally in better agreement with reference values than those with the reference orbitals. As for the difference between ROHF orbitals and canonical ROHF orbitals, canonical ROHF orbitals are more suitable for describing the electron-attached states of the reference than ROHF orbitals. The involved electron-attached states mainly contribute from one canonical ROHF orbital, while several orbitals contribute significantly to electron-attached states in some cases with ROHF orbitals. Results of the new (3,0) sector IHFSCC method using the canonical ROHF orbitals are in reasonable agreement with those with ROHF orbitals; however, their difference becomes significant when such situations show up.

### III.III Results for Molecules

Table 4 presents the equilibrium bond length (*R*_e_), harmonic frequencies (ω_e_), and adiabatic excitation energies (*T*_e_) obtained with the (3,0) IHFSCCSD method with different orbitals and different number of active orbitals for the X ^4^∑^–^ and 1^2^Π states of LiC and NaC. Results of CCSD(T), FSCCSD(2),^52^ and multireference configuration interaction (MRCI) results^74−76^ are also presented for comparison. MRCI + CPP results^74,75^ are in reasonable agreement with those of CCSD(T), while MRCI + Q results^76^ differ significantly from those of CCSD(T). This may be because of a small basis set, namely, the 6-311++G basis set,^77^ which is employed in the MRCI + Q calculations. We noted that many orbitals contribute pronouncedly to some high-lying electron-attached states with the ROHF orbitals for these two molecules. This makes it difficult to choose a larger number of active orbitals when ROHF orbitals are employed. We thus only report results of the new IHFSCC method with ROHF orbitals using 3 active orbitals in the table.

**Table 4:** Equilibrium Bond Length (Re), Harmonic Frequencies (ωe), and Adiabatic Excitation Energies (Te) with IHFSCCSD Using Different Orbitals and Active Space for the X ^4^∑^–^ and 1^2^Π States of LiC and NaC (Basis Set: aug-cc-pVTZ)

			*R*_e_ (Å)			ω_e_ (cm^–1^)			*T*_e_ (eV)		
	method	number of active orbitals	orbit1	orbit2	orbit3	orbit1	orbit2	orbit3	orbit1	orbit2	orbit3
LiH											
X ^4^∑ ^–^	IHFSCCSD	12	1.862	1.873		743	721				
	IHFSCCSD	9	1.846	1.866		772	730				
	IHFSCCSD	3	1.872	1.881	1.889	718	707	695			
	FSCCSD(2)^a^	3	1.878			698					
	MRCI + CPP^b^		1.884			691					
	CCSD(T)		1.876			706					
	IHFSCCSD	12	2.031	2.023		704	571		1.332	1.270	
1 ^2^Π	IHFSCCSD	9	1.954	2.013		682	594		1.350	1.274	
	IHFSCCSD	3	2.035	2.034	2.044	551	563	540	1.280	1.343	1.339
	FSCCSD(2)^a^	3	2.01			630			1.36		
	MRCI + CPP^c^		2.046			550			1.33		
	CCSD(T)		2.051			578			1.419		
NaC											
X ^4^∑ ^–^	IHFSCCSD	12	2.212	2.221		438	440				
	IHFSCCSD	9	2.227	2.222		423	438				
	IHFSCCSD	3	2.218	2.227	2.237	439	436	570			
	FSCCSD(2)^a^	3	2.211			475					
	MRCI + Q^d^		2.259			431					
	CCSD(T)		2.218			440					
1 ^2^Π	IHFSCCSD	12	2.456	2.394		283	337		0.959	1.109	
	IHFSCCSD	9	2.514	2.400		274	333		0.893	1.101	
	IHFSCCSD	3	2.408	2.405	2.421	335	336	318	1.124	1.212	1.201
	FSCCSD(2)^a^	3	2.335			418			1.39		
	MRCI + Q^d^		2.447			329			1.58		
	CCSD(T)		2.414			349			1.276		

a Reference (52).

b Reference (74).

c Reference (75).

d Reference (76)

One can see from Table 4 that the results of the new IHFSCC method are more sensitive to the number of active orbitals with the reference orbitals than those with canonicalized ROHF orbitals. This is especially the case with the ^2^Π state of NaC. Reasonable results can already be achieved with 3 active orbitals compared with those of CCSD(T), and further increasing the number of active orbitals does not improve results for these systems. Adiabatic excitation energies with ROHF orbitals agree well with those with canonicalized ROHF orbitals; however, the difference of harmonic frequencies for the ^2^Π states for these two molecules is pronounced. On the other hand, IHFSCC results using 3 active orbitals and reference orbitals agree reasonably well with those using canonicalized ROHF orbitals. It should be noted that adiabatic EEs with canonicalized ROHF orbitals are in better agreement with CCSD(T) results than those with reference orbitals. FSCCSD(2) can provide reliable ground-state bond lengths for these two molecules compared with CCSD(T) results, but its error on harmonic frequencies and bond lengths of excited states is larger than the present results using 3 active orbitals.

The ground state of CH and NH^+^ is ^2^Π with the electronic configuration of σ^2^ π^1^ and the σ^1^ π^2^ configuration results in the ^4^∑^–^, ^2^Δ, ^2^∑^–^, and ^2^∑^+^ states. Adiabatic excitation energies for these states of CH and NH^+^ with the (3,0) IHFSCC method using different orbitals and active spaces are listed in Table 5, together with experimental data.^71^ CCSD(T) results for the ^4^∑^–^ state are also presented for comparison. It can be seen from the table that adiabatic EEs do not change much with respect to the employed orbitals when 3 active orbitals are selected. IHFSCC results with 3 active orbitals agree reasonably well with experimental data with a difference of less than 0.25 eV. However, the results of the new IHFSCC method are rather sensitive to the number of active orbitals, even with canonicalized ROHF orbitals. Our EOM-EA-CCSD results indicate that some electron-attached states have a significant contribution from 2p1h determinants when a larger number of active orbitals is selected. This could affect the reliability of IHFSCC results. In addition, orbital mixing in some electron-attached states also occurs with canonicalized ROHF orbitals, and this could possibly also affect results obtained using canonicalized ROHF orbitals when the number of active orbitals increases. On the other hand, such a situation does not happen with the reference orbitals.

**Table 5:** Adiabatic Excitation Energies in eV with IHFSCCSD Using Different Orbitals and Active Space for NH^+^ and CH^a^

				orbit1			orbit2			orbit3		
	state	*Re* (Å)	expt.^b^	3	7	10	3	7	10	3	7	10
	^4^∑^–^	1.105	0.062(0.031)	0.123	0.376	1.757	0.118	0.116	0.044	0.120	0.193	0.266
NH^+^	^2^∑^–^	1.2511	2.752	2.925	3.206	1.842	2.970	2.127	2.232	3.084	2.661	2.614
	^2^Δ	1.1518	2.889	2.939	3.181	1.439	2.994	2.366	2.566	2.983	2.999	
	^2^∑^+^	1.1626	4.339	4.263	4.504	3.191	4.374	3.805	4.000	4.381	4.458	4.464
	^4^∑^–^	1.085	0.725(0.685)	0.535	0.645	0.428	0.482	0.500	0.518	0.447	0.440	0.637
CH	^2^Δ	1.1019	2.875	2.806	3.029	2.835	2.824	2.795	2.760	2.807	2.808	2.961
	^2^∑^–^	1.1975	3.229	3.266	3.868	4.934	3.224	3.182	3.510	3.158	3.151	3.362
	^2^∑^+^	1.1143	3.943	3.817	3.753	3.673	3.906	3.870	3.848	3.897	3.895	3.833

a CCSD(T) results are given in parentheses (basis set: aug-cc-pVTZ).

b Reference (68)

## IV Conclusions

In this paper, we report the implementation of the new IHFSCC scheme for the (3,0) sector of the Fock space to calculate triple-electron-attached states using a closed-shell reference based on our previous work on (2,0) sector IHFSCC. The intermediate Hamiltonian with the new (3,0) IHFSCC scheme depends only on *S*_2_^(1,0)^, and *S*^(2,0)^ is not required. A large active space can thus be employed to calculate TEAs with this (3,0) IHFSCC method. This (3,0) IHFSCC approach is applied to calculate triple ionization potentials and excitation energies of atoms with a p^3^ configuration and molecules with an σ^1^π^2^ configuration. Three different orbitals are employed in this work, namely, the reference orbitals, the ground ROHF orbitals of the target system, as well as the canonicalized ROHF orbitals. Effects of the active orbitals selection on results are also investigated. Our results indicate that strong orbital mixing usually occurs in the electron-attached state with ROHF orbitals, which makes a proper choice of active orbitals difficult. Results with canonicalized ROHF orbitals are generally less sensitive to the number of active orbitals than those with the reference orbitals. Reliable results are achieved with the smallest reference spaces, and canonicalized ROHF orbitals can usually provide results that agree the best with experimental data or results of other high-level methods, while increasing the number of active orbitals does not necessarily lead to improved results. The computational scaling of this new IHFSCC method is not very high, and the most expensive step is the CCSD calculation. This new (3,0) sector IHFSCC method can be applied to larger systems of physical significance such as low-lying states of triradicals.

## Appendix: Detailed Algebraic Equations for IHFSCCSD

Detailed algebraic equations for one- and two-electron parts of the intermediate Hamiltonian have already been given in ref (40). The notations employed in this work are different from those in ref (40), and these intermediates are defined as follows:

A.1

A.2

A.3

A.4

A.5

*F_me_* and *W_cmef_* in the above equations are intermediates corresponding to the transformed Hamiltonian *e*^–*T*^*He^T^* defined in the following equations:

A.6

A.7

The multiplication between the intermediate Hamiltonian and the trial vector *R^abc^* is calculated using the following equation:

A.8

*F_ce_* and *W_abef_* are also one- and two-electron intermediates of *e*^–*T*^*He^T^*, and their definition can be found in ref (78). *P*(*ab*/*c*) in the above equation is defined as

A.9

In our implementation, all of the intermediates except for *W_abef_* and *W_cmef_* are calculated explicitly and saved on the hard disk, while *W*_abef_ and *W_cmef_* are not constructed explicitly since a large amount of storage is required for saving them. In dealing with terms involving *W_abef_* and *W_cmef_*, suitable intermediates are introduced to avoid calculating them directly. The overall computational scaling for the (3,0) IHFSCC calculation is O(N^5^), where *N* represents the system size.
